# Dill (*Anethum graveolens* L.) response to sewage sludge amendment and its impact on growth and heavy metal accumulation

**DOI:** 10.1038/s41598-025-97598-9

**Published:** 2025-07-01

**Authors:** Mohamed T. Ahmed, Sulaiman A. Alrumman, Pankaj Kumar, Ebrahem M. Eid

**Affiliations:** 1 Biology Department, College of Science, King Khalid University, 61321 Abha, Saudi Arabia; 2 School of Environmental Studies, Maa Shakumbhari University, Punwarka, 247120 Saharanpur, India; 3 Botany Department, Faculty of Science, Kafrelsheikh University, Kafr El-Sheikh, 33516 Egypt; 4 Research and Development Division, Society for AgroEnvironmental Sustainability, Dehradun, 248007 India

**Keywords:** Aromatic crops, Bioaccumulation factor, Biosolid, Metal contamination, Soil fertilization, Plant sciences, Environmental sciences

## Abstract

In this study, an attempt was made to assess the effect of sewage sludge amendment (0, 10, 20, and 30 g/kg of soil) on dill plants (*Anethum graveolens* L.) cultivation using pot-scale experiments. The experiments were conducted under greenhouse conditions for 59 days and the effect of sewage sludge on soil properties as well as dill growth, productivity, and nine heavy metals (HMs) accumulation were studied. The results showed that out of the tested treatments, 10 g/kg sewage sludge application gave the best results in terms of maximum plant height (21.84 cm), number of leaves per plant (5.80), shoot biomass (0.23 g DM/plant), root biomass (0.09 g DM/plant), total biomass (0.31 g DM/plant), and absolute growth rate (0.006 g DM/day). Additionally, increased sewage sludge amendment significantly (*p* < 0.05) enhanced HM accumulation in dill roots and shoots, with the highest treatment rate (30 g/kg) consistently yielding the most pronounced effects across multiple HMs. Sewage sludge amendments reduced bioaccumulation factors (BAF) and translocation factors (TF) for Cd, Fe, Mn, Ni, and Zn in dill plants, indicating lower metal uptake and translocation to shoots with increased sludge treatment, while correlation analysis revealed that soil properties like organic matter and pH strongly influenced heavy metal bioavailability and accumulation. Overall, the present study provides insights into optimizing sewage sludge amendments for sustainable agriculture while mitigating the risks of HM contamination in dill crops.

**Supplementary Information:**

The online version contains supplementary material available at 10.1038/s41598-025-97598-9.

## Introduction

In the contemporary era of global food production, it is estimated that approximately 200 million tons of chemical fertilizers are applied to soils on an annual basis to maintain the requisite nutrient quality of soils^1^. As compared to the 1960s, fertilizer use has increased by 500% to feed the global population^2,3^. Moreover, with the global population expected to reach 9.7 billion by 2050, fertilizer demand is projected to increase by 10–15% to maintain food security, particularly in developing nations^4,5^. Nitrogen-based fertilizers account for approximately 60% of the total global consumption of fertilizers, with phosphorus and potassium fertilizers accounting for the remaining proportion^6,7^. As per an efficiency gap study, it is reported that only 40–50% of fertilizers are consumed by plants while the rest is lost in the environment through the processes of leaching, runoff, and volatilization^8^. Also, an estimated 33% of the world’s arable land is degraded due to improper or overuse of fertilizers, leading to nutrient imbalances in the soil^9^. Since fertilizers play an important role in global food security, they are crucial for maintaining soil fertility^10^.

Sewage sludge, also known as biosolids, is a byproduct of the wastewater treatment process that is processed and treated to become a nutrient-rich material used as a biofertilizer^11,12^. Farmers have been using sewage sludge as a sustainable biofertilizer that supports the approach of a circular economy by recycling the waste generated by wastewater treatment plants into productive agricultural use^13^. Sewage sludge contains essential nutrients such as nitrogen, phosphorus, potassium, and microelements, which can help in maintaining soil fertility^14^. It is also rich in organic matter which improves water retention, aeration, and microbial activities^15^. The incorporation of sewage sludge into soil has the potential to facilitate carbon sequestration, thereby contributing to the mitigation of climate change through the reduction of atmospheric carbon dioxide levels^16,17^. Sewage sludge has been commonly used as economic fertilizer for several crops such as wheat (*Triticum aestivum* L.)^18^, rice (*Oryza sativa* L.)^19^, tomato (*Solanum lycopersicum* L.)^20^, potato (*S. tuberosum* L.)^21^, eggplant (*S. melongena* L.)^22^, marigold (*Tagetes erecta* L.)^23^, luffa (*Luffa acutangula* (L.) Roxb)^24^, etc. However, there have been concerns regarding the accumulation of potentially toxic heavy metals in cultivated crops grown under the long-term application of sewage sludge^25^. For instance, sewage sludge application resulted in high levels of monitored heavy metals in guar (*Cyamopsis tetragonoloba* (L.) Taub.)^26^, *T. aestivum*^27^, maize (*Zea mays* L.)^28^, and spinach (*Spinacia oleracea* L.)^29^ plants. Therefore, it is necessary to properly control and regularly monitor the levels of these heavy metals in cultivated crops and their products^30^.

Dill (*Anethum graveolens* L.) is an annual herbaceous plant in the Apiaceae family, widely recognized for its culinary and medicinal uses^31^. Dill is native to southwestern Asia and the Mediterranean, but it is now recognized globally due to its aromatic flavor and therapeutic properties^32^. Dill plants are characterized by their slender stems, delicate feathery leaves, and umbels of small yellow flowers^33,34^. The plant reaches a height of 40–60 cm and thrives in temperate climates with well-drained soils and full-day sunlight^35^. In culinary uses, both the leaves (commonly referred to as dill weed) and seeds are widely used^36^. The leaves impart a subtle, slightly sweet, and tangy flavor to dishes, making them a popular ingredient in salads, soups, fish dishes, and pickling^37^. Dill seeds, which have a more pungent flavor profile, are utilized as a spice in a multitude of culinary traditions, particularly in bread-making and as a seasoning for vegetables and meats^38^. Dill plants have a long history of use in traditional medicine beyond their culinary applications^31^. It is a rich source of essential oils, including carvone, limonene, and dillapiole, which contribute to its carminative, digestive, and anti-inflammatory properties^33^. Dill has a longstanding tradition of use in the treatment of digestive ailments, including indigestion, bloating, and flatulence^39^. Its seeds have also been employed as a mild diuretic and to promote lactation in nursing mothers^40^.

While sewage sludge has been studied as a soil amendment for various staple and horticultural crops (like wheat, rice, maize, and vegetables), its application and associated risks in aromatic herbs, particularly dill, remain underexplored. Given the widespread cultivation of dill plants across the Mediterranean, Middle Eastern, and South Asian regions due to their utility, this study proposes that applying sewage sludge at different levels will enhance soil fertility and improve dill plant productivity. To date, no research has explored the use of sewage sludge as an organic amendment in dill cultivation. Thus, this study aims to evaluate the impact of varying doses of sewage sludge on dill growth, productivity, and potential heavy metal accumulation. The findings contribute valuable insights into the safe and sustainable application of sewage sludge for dill production.

## Material and methods

### Plant materials, sewage sludge treatments, and experimental design

Verified dill seeds (ZORZI, HORTUS SEMENTI srl, Longiano, Italy) were procured from a local market in Abha City, Saudi Arabia. The soil utilized for the experiment in the cultivated field was a coarse sandy loam, specifically Typic Torriorthents as classified by the Soil Survey Staff^41^. This soil was collected from neighboring areas recently reclaimed, at a depth of 0–20 cm (with coordinates: Latitude: 18.2407, Longitude: 42.5709). The sewage sludge used in the study was obtained from the Abha City Municipal Wastewater Treatment Plant (with coordinates: Latitude: 18.2331, Longitude: 42.5212). Both the cultivated field soil and sewage sludge samples were air-dried for a period of two weeks, ground, and sieved through a 2 mm mesh. The experiment took place in the greenhouse of the Biology Department at King Khalid University. Prior to the main experiment, a preliminary experiment was conducted to determine the appropriate mixing ratio of sewage sludge with the cultivated field soil. The rates tested were 0 g/kg (as a control), 10, 20, and 30 g/kg. Each treatment consisted of nine (9 L) plastic pots, each filled with 4 kg of the specific treatment and 20 seeds. The experimental units were arranged in a completely randomized design. The plants were grown for a duration of 59 days, starting from December 18, 2022, in the greenhouse, following the natural day-night cycle. Irrigation was carried out using tap water as needed to maintain a water content of 40–50% in each pot. Manual weeding was conducted as required, and after 15 days, the plants were manually thinned to five plants per pot.

### Plant morphology and biomass

The dill plants were harvested on February 14, 2023. A measuring tape was used to measure the heights of the shoots, and the number of leaves per plant was counted. All plant materials underwent a thorough washing process under running water, followed by rinsing with deionized water. The materials were then divided into roots and shoots. The partitioned plant materials were dried at 60 °C until they reached a constant weight, and their masses were recorded. Subsequently, the samples were ground using a plastic mill and stored for future analyses. The total biomass, which is the sum of shoot and root biomass, was calculated. The absolute relative growth rate (RGR) was determined according to the method described by Radford^42^:

$${\text{RGR }}\left( {{\text{g}}\;{\text{DM}}/{\text{ind}}./{\text{day}}} \right) \, = \, \left( {W_{2} {-}W_{1} } \right)/\left( {t_{2} {-}t_{1} } \right)$$

where *W*_*1*_ and *W*_*2*_ represent the total biomasses (g DM/ind.) at times (days) *t*_*1*_ and *t*_*2*_, respectively.

### Sample analysis

After the termination of experiments, soil samples were collected from each replicate. These samples were air-dried for a period of 2 weeks, and then ground and sieved through a 2 mm mesh. The soil samples from all treatments, as well as the sewage sludge and cultivated field soil, were subjected to analysis to determine their OM contents using the loss-on-ignition method at 550 °C for 2 h^43^. Additionally, nitrogen (N) content was analyzed using a CHN Elemental Analyzer (Yanako CHN Corder MT-5 and Auto Sampler MTA-3, Yanako Co., Ltd., Kyoto, Japan). The electrical conductivity (EC) and pH were measured in 1:5 soil–water extracts^44^. To determine the contents of phosphorus (P), potassium (K), and HMs, 0.5–1.0 g of each soil, SS, cultivated field soil, and plant sample (for HMs only) was digested using a tri-acid mix digestion method (HNO_3_:H_2_SO_4_:HClO_4_; 5:1:1, *v*/*v*/*v*). A microwave sample preparation system (PerkinElmer Titan MPS, PerkinElmer Inc., USA) was used for the digestion process. Blank samples were included to ensure the accuracy and precision of the digestion procedure and subsequent analyses. The concentrations of nine HMs (cadmium: Cd, cobalt: Co, chromium: Cr, copper: Cu, iron: Fe, manganese: Mn, nickel: Ni, lead: Pb, and zinc: Zn) and K were determined using inductively coupled plasma optical emission spectrometry (ICP-OES) (Thermo Scientific iCAP 7000 Plus Series; Thermo Fisher Scientific, Massachusetts, USA) following the method described by Allen^44^. The detection limits for these elements (in µg/L) were as follows: 6.0 for Ni; 2.0 for Co, Cr, Cu; 1.0 for Fe, Pb, Zn; 0.3 for Mn; 0.1 for Cd; and 0.2 for K. Phosphorus (P) content was determined using a spectrophotometer (CECIL CE 1021, Cecil Instruments Limited, Cambridge, UK) utilizing the ammonium-molybdate method. The instrument settings and operational conditions followed the manufacturer’s specifications, and standard solutions with known element contents were prepared to calibrate the system.

### Quality assurance and quality control

To validate the accuracy of the HM determinations, a certified reference material (SRM 1573a, tomato leaves) was employed. This reference material underwent the same digestion and analysis methods as the dill samples. The HM digestions and measurements were conducted in triplicate. Accuracy was assessed by comparing the measured content with the certified value, and the outcome was expressed as a percentage. The recovery rates of HMs varied from 95.80 to 104.80%.

### Data analyses

The bioaccumulation factor (BAF) is employed to assess the ability of dill plants to accumulate HMs in their roots, while the translocation factor (TF) is used to estimate the potential of dill plants to transfer HMs from the roots to the aerial shoot tissues. The calculations for BAF and TF were performed using following the method described by Eid and Shaltout^45^:

$${\text{BAF }} = \frac{{{\text{Heavy }}\;{\text{metal }}\,{\text{content }}\;{\text{in }}\,{\text{the }}\;{\text{root (mg/kg) }}}}{{{\text{Heavy }}\;{\text{metal }}\;{\text{content }}\;{\text{in }}\;{\text{the }}\;{\text{soil (mg/kg)}}}}$$

while

$${\text{ TF}} = \frac{{{\text{Heavy }}\;{\text{metal }}\;{\text{content }}\;{\text{in }}\;{\text{the }}\;{\text{shoot (mg/kg) }}}}{{{\text{Heavy }}\;{\text{metal }}\;{\text{content}}\;{\text{ in }}\;{\text{the }}\;{\text{root (mg/kg) }}}}$$

Before performing the analysis, the data were assessed for homogeneity of variance using Levene’s test and for normality of distribution using Shapiro–Wilk’s test. To assess the impact of different sewage sludge amendment rates on soil properties, dill growth (biomass and morphology), heavy metal (HM) accumulation in dill tissues, and the transfer of HMs from soil to plant (bioaccumulation and translocation factors), pairwise comparisons between each treatment and the control were conducted using Student’s *t*-test. Additionally, a Student’s *t*-test was applied to compare the chemical properties of cultivated field soil and sewage sludge. Pearson correlation coefficients (*r*) were calculated to assess the relationship between soil characteristics and HM contents in dill tissues (shoots and roots). For all statistical analyses, SPSS 23 software^46^ was employed.

## Results and discussion

### Properties of sewage sludge and soil used for dill cultivation

As shown in Table S1, the chemical properties of cultivated field soil and sewage sludge show significant differences. Specifically, the cultivated soil exhibits an alkaline pH (8.68 ± 0.02), while the sewage sludge has a slightly acidic pH (7.57 ± 0.01). EC of the sewage sludge is higher than that of the soil, which may affect plant nutrient uptake. Dill plants can be cultivated in a pH range of slightly acidic to neutral (5.5–7.5) however, a lower EC is preferred for better root development^47^. Moreover, OM is substantially higher in sewage sludge (68.80 ± 0.35%) compared to the field soil (0.91 ± 0.10%), indicating the high organic content. Also, N, K, and P contents are significantly high in both mediums, with sewage sludge having higher N but lower P levels compared to the soil. On the other hand, HM analysis shows that both samples fall within permissible limits for Cd, Co, Cr, Cu, Ni, Pb, and Zn as per The Council of the European Communities^48^, though some metals, such as Pb and Zn, are substantially higher in sewage sludge. Herein, the cultivated soil exceeds the normal limit for Cr and Fe concentrations as per Kabata-Pendias^49^. These results suggest that sewage sludge is nutrient-rich but contains higher metal concentrations, necessitating careful management in agricultural applications through controlled dose applications.

**Fig. 1:**
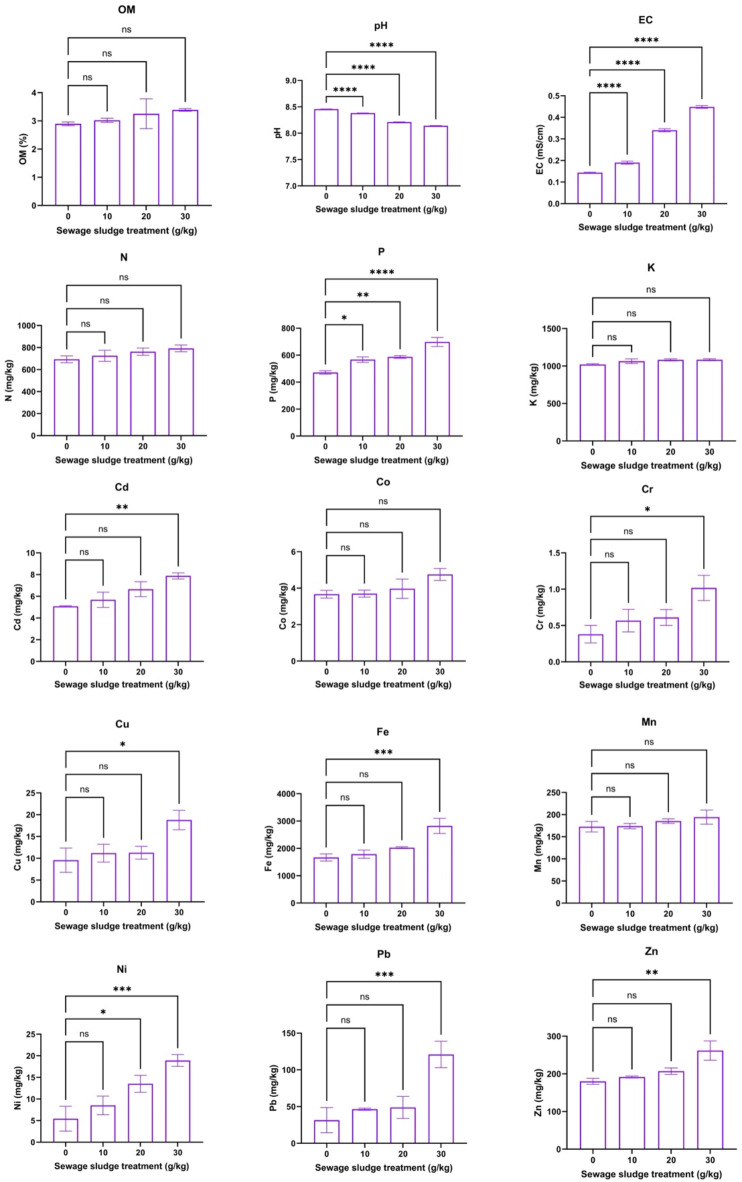
Selected chemical properties of soil at different sewage sludge amendment rates after harvesting dill plants (*Anethum graveolens* L.) that had been grown for 59 days (means ± standard error, *n* = 9). Student’s *t*-test comparisons were displayed on the graphs. *: *p* < 0.05; **: *p* < 0.01; ***: *p* < 0.001; ****: *p* < 0.0001; *ns*: not significant (i.e., *p* > 0.05).

Figure 1 shows the effects of varying sewage sludge amendment rates (0, 10, 20, and 30 g/kg) on the chemical properties of soil after 59 days of dill cultivation. Statistically significant changes (*p* < 0.05) were observed in several soil parameters following the experiment. Soil EC increased while soil pH decreased proportionally with higher sludge application rates. However, N and OM remained largely unaffected, while P exhibited a consistent increase. HM concentrations in the soil also increased as the sewage sludge dosage increased. Therefore, in the present study, the soil properties are in line with those suggested by previous studies on dill cultivation^47,50^. Thus, a significant increase in soil EC and heavy metal concentrations with increased sludge amendment rates suggests the necessity to adopt soil pollution management strategies. Additionally, the increasing trend of soil P levels indicates that sludge can increase the bioavailability of nutrients, which can lead to better dill productivity. However, the drop in soil pH with increasing sludge application rates is significant, and its implications for the availability of nutrients and plant health should be carefully monitored.

### Effect of sewage sludge on dill growth and productivity

**Fig. 2:**
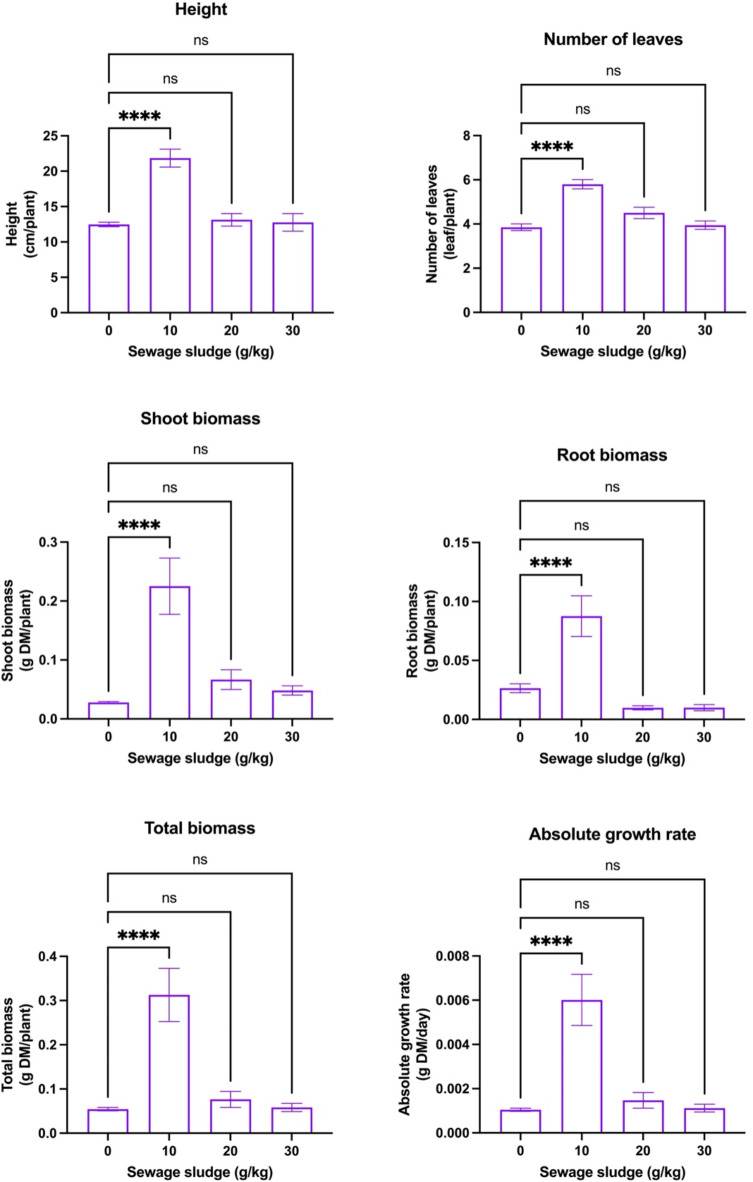
Effects of different sewage sludge amendment rates on the morphometric and biomass parameters of dill plants (*Anethum graveolens* L.) harvested after 59 days (means ± standard error, *n* = 45). Student’s *t*-test comparisons were displayed on the graphs. ****: *p* < 0.0001; *ns*: not significant (i.e., *p* > 0.05).

Figure 2 shows the effects of different sewage sludge amendment rates on the selected morphometric and biomass parameters of dill plants. It was evident that the plant height of dill plants (21.84 cm) was significantly (*p* < 0.05) increased by 1.75-fold in 10 g/kg sewage sludge treatment as compared to control treatment. However, it was found non-significantly (*p* > 0.05) affected in further higher dose treatments. A similar trend was observed for the number of plant leaves with maximum values observed in the 10 g/kg treatment i.e., 5.80 leaves per plant as compared to 3.85 in the control treatment. Similarly, shoot, root, and total biomass were also reported highest in 10 g/kg treatment i.e., 0.23, 0.09, and 0.31 g DM/plant, respectively. The same treatment showed the highest relative plant growth rate of 0.006 g DM/day depicting better survival of dill plants during its cultivation. In this study, sewage sludge application showed both positive and negative effects on the growth parameters of dill plants, depending on the application rate of the sludge. Sewage sludge being rich in NPK nutrients, which are vital for plant growth could have resulted in enhanced growth, better leaf development, and increased biomass in dill plants.

Previous reports have shown that sewage sludge adds OM to the soil, improving soil structure, water retention, and aeration. Increased OM enhances root growth and development, leading to a healthier plant with greater shoot and root biomass^51^. OM also stimulates soil microbial activity, which helps decompose organic compounds and makes nutrients more available to dill plants. However, excessive addition of sewage sludge could negatively affect soil health and structure sewage sludge often contains heavy metals like Cd, Pb, Hg, and Zn. When applied in excess, these metals can accumulate in the soil and be taken up by plants, leading to phytotoxicity^52^. In plants, this can result in stunted growth, leaf chlorosis, and reduced biomass due to inhibited enzyme activities and disrupted physiological processes^53^. Thus, the observed reduction in plant growth in the present study, in response to the addition of sewage sludge treatments over 10 g/kg, may be attributed to induced soil toxicity. Recent studies by Elsayed et al.^54^ reported maximum significant (*p* < 0.05) improvements in yield and biochemical constituents of dill when grown using chicken manure as an organic amendment. Another study by Zende Bad et al.^55^ studied the effects of cow manure and vermicompost in combination with chemical fertilizer on dill and found that yield was positively influenced in multiple cuttings. Similarly, Pirdashti et al.^56^ found that municipal application of waste compost and *Trichoderma* spp. showed a significant increase in dill production and microelement uptake. Overall, the study indicates that a moderate sewage sludge amendment rate of 10 g/kg optimally enhances dill growth and productivity by improving nutrient availability and biomass accumulation. However, higher doses may induce soil toxicity due to heavy metal accumulation, emphasizing the need for controlled sludge application to ensure sustainable crop production.

### Effect of sewage sludge on heavy metal contents in dill tissues

**Fig. 3:**
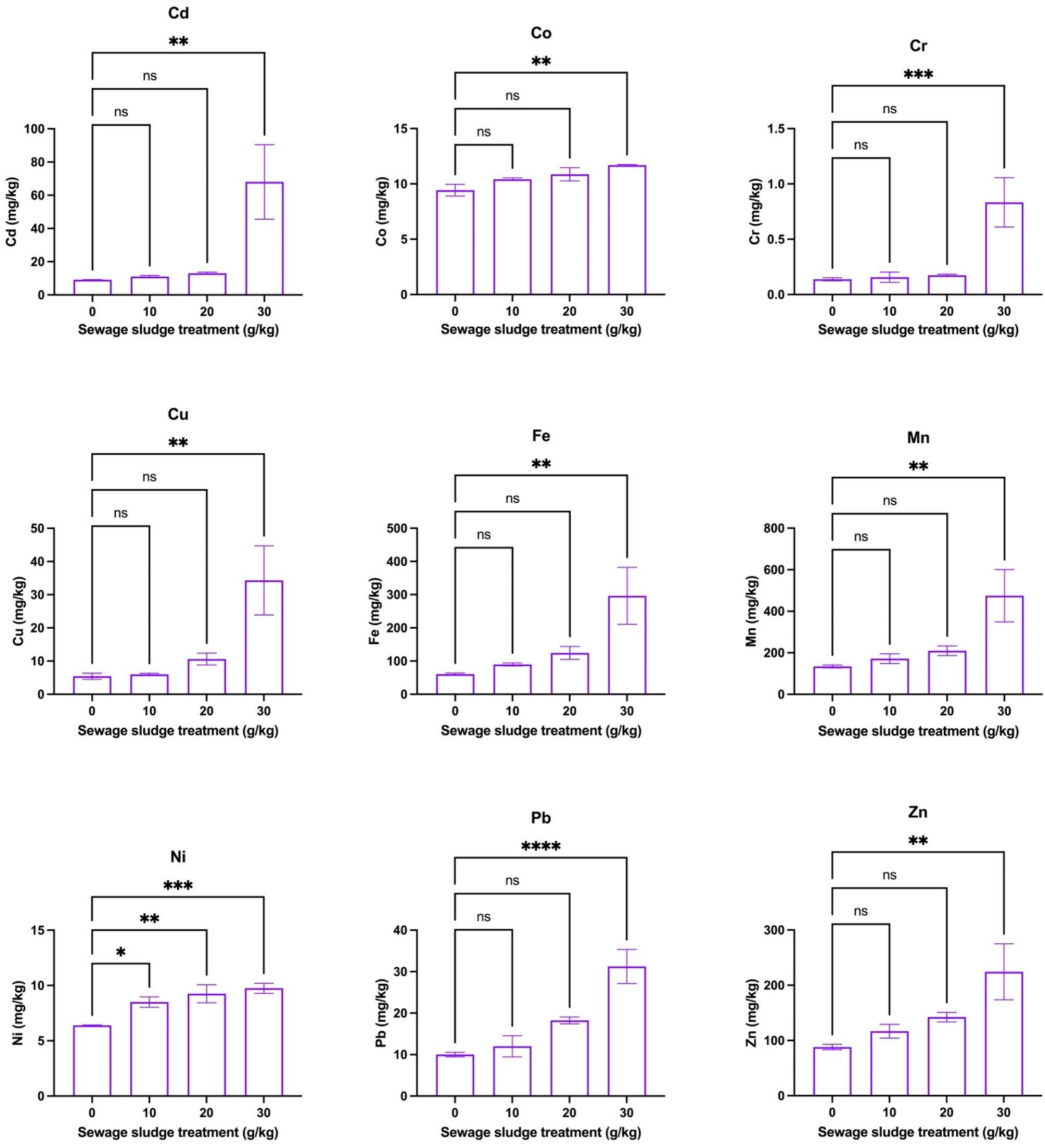
Effects of different amendment rates of sewage sludge on metal contents (mg/kg) in roots of dill plants (*Anethum graveolens* L.) that were harvested after 59 days (means ± standard error, *n* = 9). Student’s *t*-test comparisons were displayed on the graphs. *: *p* < 0.05; **: *p* < 0.01; ***: *p* < 0.001; ****: *p* < 0.0001; *ns*: not significant (i.e., *p* > 0.05).

**Fig. 4:**
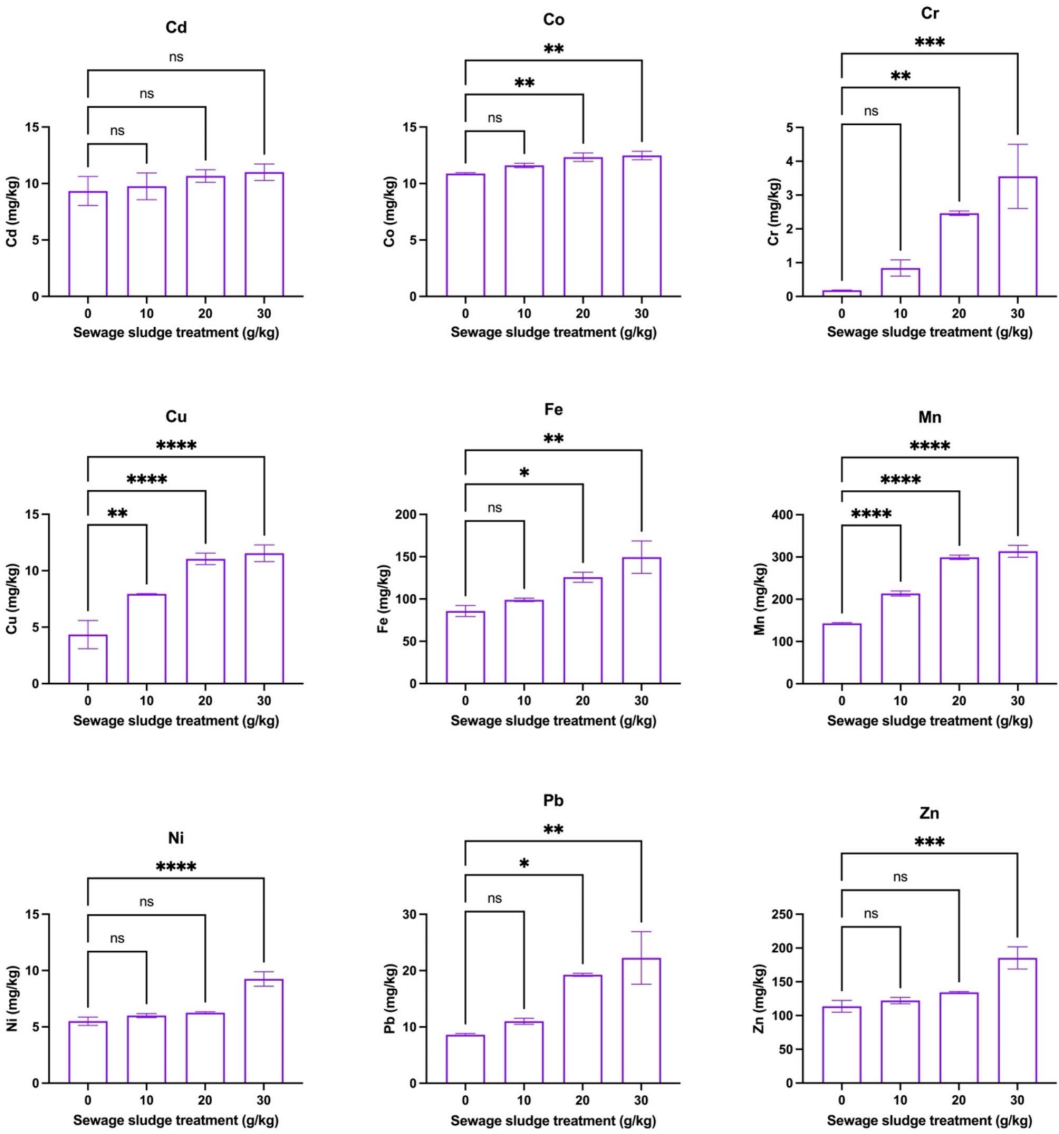
Effects of different amendment rates of sewage sludge on metal contents (mg/kg) in shoots of dill plants (*Anethum graveolens* L.) that were harvested after 59 days (means ± standard error, *n* = 9). Student’s *t*-test comparisons were displayed on the graphs. *: *p* < 0.05; **: *p* < 0.01; ***: *p* < 0.001; ****: *p* < 0.0001; *ns*: not significant (i.e., *p* > 0.05).

Figure 3 shows the effects of different amendment rates of sewage sludge on HM contents (mg/kg) in the roots of dill plants after 59 days of growth. The findings showed a significant increase in HM concentrations was observed with increased sludge amendment for most metals. Specifically, Cd (68.00 mg/kg), Co (11.69 mg/kg), Cr (0.83 mg/kg), Cu (34.32 mg/kg), Fe (296.48 mg/kg), Mn (474.83 mg/kg), Pb (31.25 mg/kg), and Zn (224.27 mg/kg) showed maximum significant concentration at 30 g/kg sewage sludge (*p* < 0.05), while lower treatments did not show significant differences. In addition, Ni had a dose-dependent response to sewage sludge amendments (at 10 g/kg: 8.50 mg/kg, at 20 g/kg: 9.25 mg/kg, at 30 g/kg: 9.75 mg/kg). These patterns showed that higher amendment rates of sewage sludge more effectively increased metal uptake by dill roots. On the other hand, Fig. 4 depicts the impact of various sewage sludge amendment rates on HM concentrations in the shoots of dill plants after 59 days. In this case, Cd levels (9.33–11.00 mg/kg) in the shoots remained unaffected across 0–30 g/kg sewage sludge treatments (*p* > 0.05). Also, Co concentrations increased significantly at 20 and 30 g/kg (*p* < 0.05) i.e., 12.33 and 12.48 mg/kg, respectively. Similarly, Cr content was highly significant at both 20 g/kg (2.46 mg/kg) and 30 g/kg (3.55 mg/kg), compared to the control (0.18 mg/kg). A similar pattern was observed for Cu levels which showed a sharp increase from 10 to 30 g/kg. Also, Fe, concentrations increased significantly at 20 to 30 g/kg (*p* < 0.05) with values of 125.68 and 149.44 mg/kg while Mn, Ni, Pb, and Zn exhibited substantial increases in content at 30 g/kg (*p* < 0.05), respectively. Overall, results indicated that increasing sewage sludge treatment enhances HM accumulation in dill plant roots, with the highest treatment (30 g/kg) consistently showing the most pronounced effects.

It is evident that OM can enhance HM bioavailability by improving soil structure and cation exchange capacity^57^ which facilitates the uptake of metals by plant roots. In this study, higher loading rates of sewage sludge resulted in higher concentrations of HMs in the soil, creating a dose-dependent relationship where plants absorb more metals with increasing sludge applications. It might be possible that Cd and Co have high mobility and affinity for OM which resulted in their maximum uptake by dill plants in higher treatments. Also, other HMs such as Cr, Ni, Cu, and Pb showed dose-dependent increases, suggesting that these HMs are available in soluble forms, which promotes their translocation to roots and shoots. However, certain HMs, such as Cd in the shoots, did not increase across sludge treatments, indicating possible selective metal uptake mechanisms within the plants or limitations in translocation from roots to shoots^58^. The differential heavy metal uptake and translocation in dill plants under varying sewage sludge treatments can be attributed to multiple pathways, including root surface adsorption, apoplastic and symplastic transport, and metal sequestration in vacuoles. These mechanisms are influenced by soil properties like pH and OM, affecting metal bioavailability and plant uptake^57^. Factors such as soil pH, OM content, and HM speciation significantly influence the mechanisms of metal uptake and translocation in plants. Lower pH enhances metal solubility, while higher OM can immobilize metals. These factors collectively affect metal bioavailability, root absorption efficiency, and subsequent translocation within plant tissues. Previous studies have shown that dill cultivation in Zn–Cu HM contaminated soil areas of Plovdiv, Bulgaria. They reported that dill plants significantly accumulated selected HMs such as Cd, Pb, Cu, Mn, and Zn in dill tissues^59^. Similarly, Zheljazkov and Warman^60^. also reported that the application of compost having high Cu levels for dill fertilization resulted in higher HM accumulation by dill and peppermint plants. A study by Caunii et al.^61^ also depicted that soil contamination with HMs resulted in their higher mobility in dill plants with maximum contents of Cu in both roots and shoot tissues. Thus, these studies also indicated that soil HM levels exert proportional effects on the uptake of these elements by dill plant tissues. The results showed that higher sewage sludge amendment rates, particularly 30 g/kg, significantly increased heavy metal accumulation in dill roots and shoots. The dose-dependent uptake, especially for Cd, Co, Cr, and Cu, shows the critical need for controlled sludge application to minimize potential phytotoxicity and ensure safe crop production.

### Bioaccumulation, translocation, and correlation studies

**Fig. 5:**
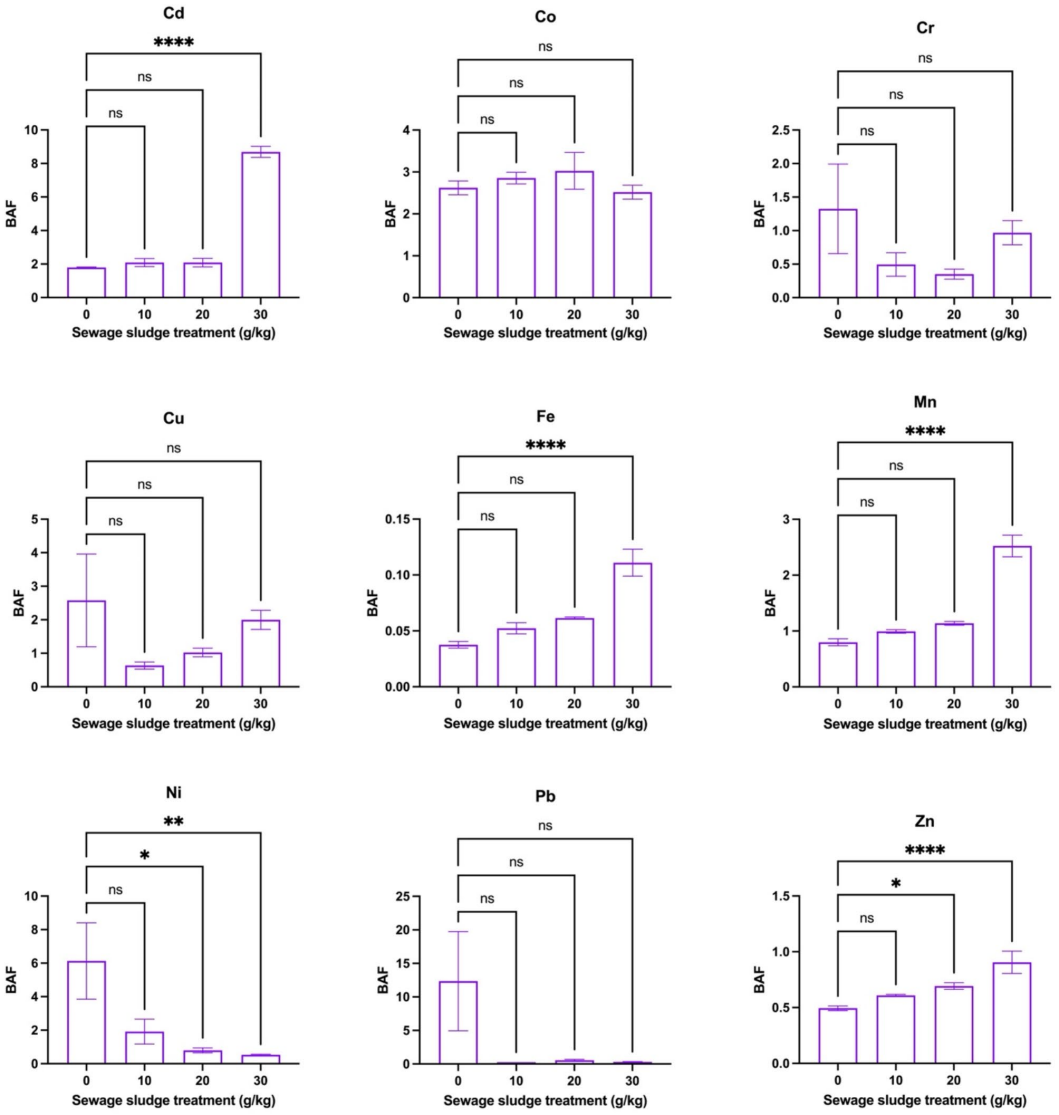
Bioaccumulation factors (BAFs), from soil to roots, of metals in dill plants (*Anethum graveolens* L.) grown in soil with different sewage sludge amendment rates (means ± standard error, *n* = 9). Student’s *t*-test comparisons were displayed on the graphs. *: *p* < 0.05; **: *p* < 0.01; ****: *p* < 0.0001; *ns*: not significant (i.e., *p* > 0.05).

**Fig. 6:**
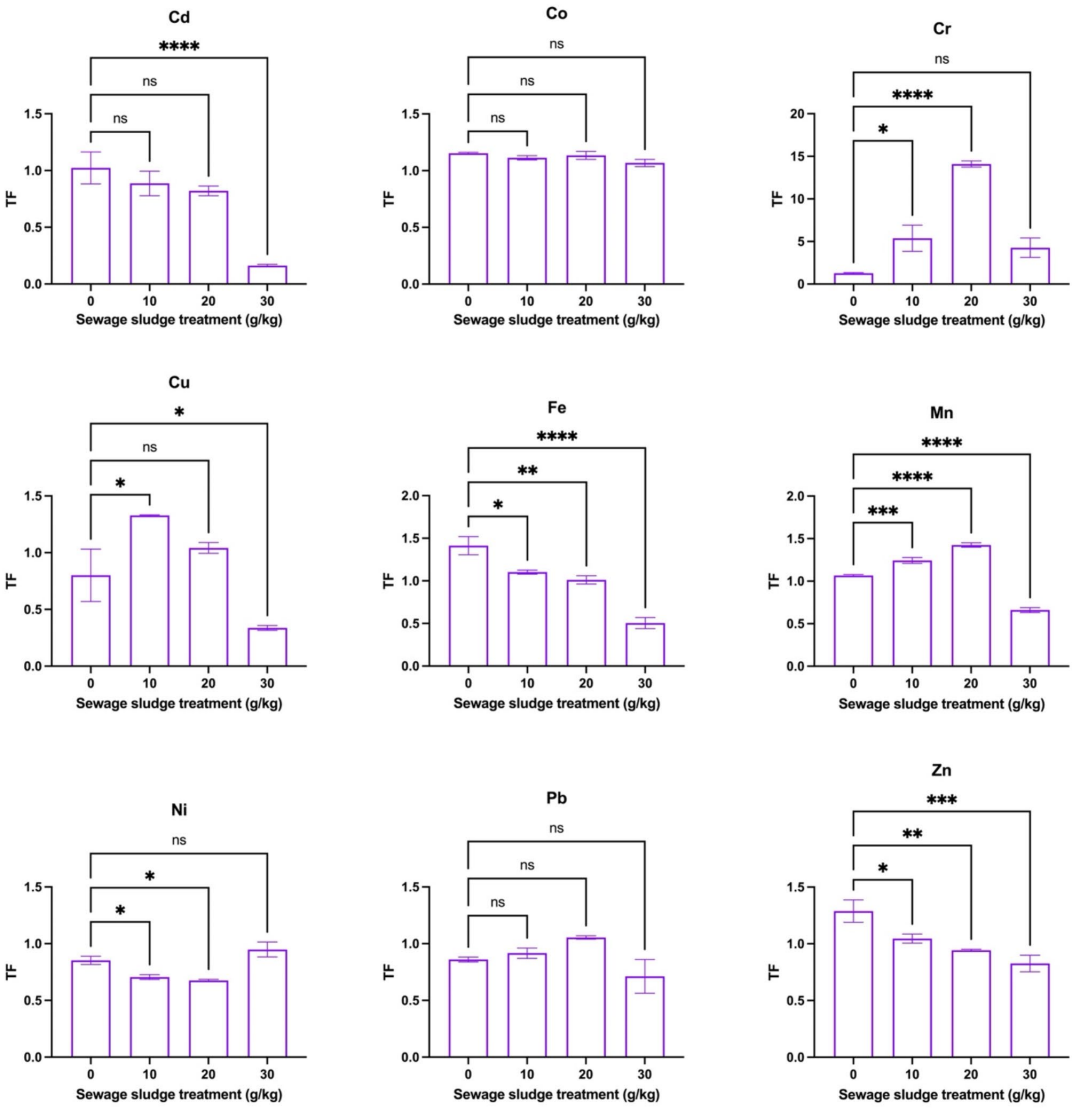
Translocation factors (TFs), from roots to shoots, of metals in dill plants (*Anethum graveolens* L.) grown in soil with different sewage sludge amendment rates (means ± standard error, *n* = 9). Student’s *t*-test comparisons were displayed on the graphs. *: *p* < 0.05; **: *p* < 0.01; ***: *p* < 0.001; ****: *p* < 0.0001; *ns*: not significant (i.e., *p* > 0.05).

**Fig. 7:**
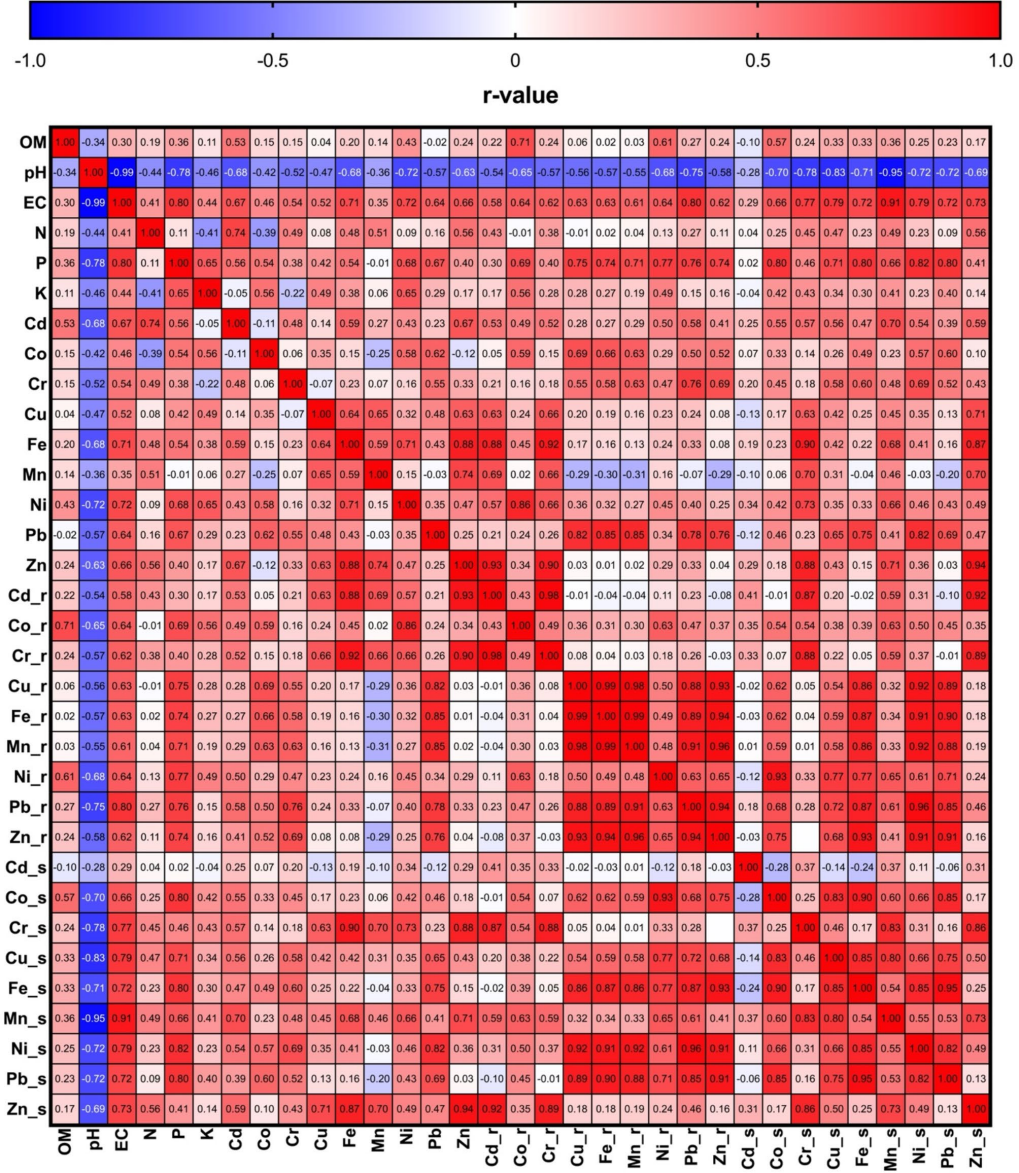
Pearson correlation coefficient (*r*-values, *n* = 36) between nine heavy metals in dill (*Anethum graveolens* L.) shoots and roots and their contents in the soil-sludge mixture.

Figure 5 shows BAF trends for selected HMs in dill plants grown in sewage sludge-amended soil. The findings showed that significant increases in values of BAF were observed for Cd (1.79 to 8.69), Fe (0.04 to 0.11), Mn (0.80 to 2.52), and Zn (0.49 to 0.91) with increasing sewage sludge treatment, indicating elevated HM uptake by roots. Specifically, for Cd and Zn, this increment was highly significant (*p* < 0.05), while Ni showed a dose-dependent increase (*p* < 0.05). However, Co, Cu, Pb, and Cr showed no significant changes across treatments, suggesting sewage sludge had limited influence on their bioaccumulation. Similarly, Fig. 6 depicts that TFs had significant variations in the movement of HMs from roots to shoots in dill plants under different sewage sludge treatments. For Cd (1.02 to 0.16), Fe (1.41 to 0.50), Mn (1.07 to 0.66), and Zn (1.29 to 0.83), TFs significantly decreased with increased sludge application, indicating inhibited metal translocation to shoots at higher sludge rates. Herein, the reduction was highly significant for Cd, Fe, Mn, and Cr (*p* < 0.05). On the other hand, Co, Cu, Pb, and Ni exhibited non-significant or inconsistent responses to sludge treatments, showing variability in their mobility within the plant. On the other hand, Fig. 7 shows the Pearson correlation matrix (*r*-values) between HM concentrations in dill shoots and roots, and the corresponding levels in the soil-sludge mixture. Herein, strong positive correlations (*r* > 0.7) were observed between soil HM concentrations and their accumulation in both roots and shoots, indicating a direct relationship between soil contamination and plant uptake. However, some negative correlations such as pH with certain metals like Cd and Zn indicated a reduced bioavailability with higher soil pH. Thus, soil properties had a strong influence on HM bioaccumulation in plants, with OM and pH being significant parameters in metal mobility and uptake. In this study, a high sludge treatment (30 g/kg) led to increased HM accumulation in plant tissues but decreased BAF and TF, which might be attributed to metal immobilization dynamics and physiological responses of dill plants. At higher sludge levels, although soil HM concentrations increased, several factors like high OM and changes in pH reduced metal bioavailability and mobility, thus decreasing TFs. Additionally, plants may activate defense mechanisms such as metal sequestration in root cell walls or vacuoles, limiting translocation to shoots. Hence, while total HM uptake in roots increased, the restricted mobility led to reduced BAF and TF values.

These results are in line with those reported by Caunii et al.^61^ on the effect of soil contamination on BAF of selected HMs in dill plants. They reported that Cu and Pb showed the highest BAF values of 13.5 and 3.76, respectively. Jalali and Meyari^62^ also found that the TF of Cd and Pb was high in dill designating it as a hyperaccumulator plant cultivated in western Iran. Additionally, Alhogbi et al.^63^ recently reported that the TF of selected HMs (Pb, Ni, Zn, Fe, Cr, and Cu) in dill plants was significantly affected by the type of irrigation water, subsequently influencing HMs levels in the soil. Overall, sewage sludge treatments significantly reduced the bioaccumulation and translocation of HMs in dill, with soil properties like OM and pH further influencing HM mobility and uptake, consistent with previous studies. Thus, the results showed that while sewage sludge amendments enhance HM bioaccumulation in dill roots, they significantly inhibit translocation to shoots, particularly for Cd, Fe, Mn, and Zn. Strong soil–plant correlations indicate the key role of soil properties, especially OM and pH, in governing metal mobility and plant uptake.

## Conclusion

The objective of this study was to evaluate the influence of sewage sludge amendment on the growth, productivity, and HM accumulation in dill plants. The findings indicated that sewage sludge application resulted in a notable enhancement of soil nutrient contents, thereby promoting increased dill productivity. However, the application of sewage sludge above 10 g/kg was not found to be a viable option, as evidenced by the observed plant growth attributes. Additionally, the HM contents in dill tissues (roots and shoots) exhibited a consistent increase with the application of increasing doses of sewage sludge. The bioaccumulation, translocation, and correlation studies indicated that higher sewage sludge applications could potentially be toxic to dill plants, resulting in the accumulation of elevated levels of HMs in the plants, which may make them unsuitable for consumption. Further studies are recommended for understanding the mechanisms and uptake modeling of HMs by dill plants under sewage sludge treatment, while also characterizing the associated health risks. Also, further research should focus on developing strategies to mitigate heavy metal uptake in dill plants grown in sludge-amended soils while further investigating the environmental risks of the sludge application rate (10 g/kg and above). Additionally, exploring the potential use of dill in phytoremediation and assessing the long-term environmental impacts of sewage sludge application can provide a better understanding of sustainable agricultural practices.

## Electronic supplementary material

Below is the link to the electronic supplementary material.

Supplementary Material 1

## Data Availability

Data will be made available on reasonable request to the corresponding author.

## References

[1] Timsina, J. Can organic sources of nutrients increase crop yields to meet global food demand?. *Agronomy* **8**, 214 (2018).

[2] Shiferaw, B. et al. Crops that feed the world 10. Past successes and future challenges to the role played by wheat in global food security. *Food Secur.* **5**, 291–317 (2013).

[3] Fuglie, K. O., Morgan, S. & Jelliffe, J. *World Agricultural Production, Resource Use, and Productivity, 1961–2020*. www.ers.usda.gov (2024).

[4] Ferreira, A. C. S., Cruz, R. C., Rosa, C. Q., de Fátima, Â. & Modolo, L. V. Reaching food security: harnessing urease inhibitors to meet the challenges of growing global population. In *Ureases* 359–373 (2024). 10.1016/B978-0-323-91800-8.00009-6.

[5] Gao, Y., Dong, K. & Yue, Y. Projecting global fertilizer consumption under shared socioeconomic pathway (SSP) scenarios using an approach of ensemble machine learning. *Sci. Total Environ.* **912**, 169130 (2024). 38070571 10.1016/j.scitotenv.2023.169130

[6] Adenäuer, M., Laget, E. & Cluff, M. Fertile futures: Scenario analysis on the interconnected dynamics of fertiliser and agricultural markets. *OECD Food Agric. Fish. Pap.* **207**, 1037 (2024).

[7] Lim, J., Fernández, C. A., Lee, S. W. & Hatzell, M. C. Ammonia and nitric acid demands for fertilizer use in 2050. *ACS Energy Lett.* **6**, 3676–3685 (2021).

[8] Bibi, S., Saifullah, A. N. & Dahlawi, S. Environmental impacts of nitrogen use in agriculture, nitrate leaching and mitigation strategies. In *Soil Science: Agricultural and Environmental Prospectives* (eds Hakeem, K. R. et al.) 131–157 (Springer, 2016). 10.1007/978-3-319-34451-5_6.

[9] Abebaw, W. A. Review on impacts of land degradation on agricultural production in Ethiopia. *J. Resour. Dev. Manag.* **57**, 21–29 (2019).

[10] Lin, L. et al. Analysis of mineral phases in heavy-metal hazardous waste under the interdisciplinary scope of data science and chemistry. *Prog. Chem.* 211120. 10.7536/PC211120 (2021).

[11] Taron, A., Singh, S., Drechsel, P., Ravishankar, C. & Ulrich, A. *Sewage Sludge: A Review of Business Models for Resource Recovery and Reuse*. *Resource Recovery and Reuse* vol. 23 https://hdl.handle.net/10568/135464 (2023).

[12] Wang, Y. et al. Unraveling how Fe-Mn modified biochar mitigates sulfamonomethoxine in soil water: The activated biodegradation and hydroxyl radicals formation. *J. Hazard. Mater.* **465**, 133490. 10.1016/j.jhazmat.2024.133490 (2024). 38228002 10.1016/j.jhazmat.2024.133490

[13] Chojnacka, K. et al. Practical aspects of biowastes conversion to fertilizers. *Biomass Convers. Biorefin.* **14**, 1515–1533 (2024).

[14] Mohammad, M. J. & Athamneh, B. M. Changes in soil fertility and plant uptake of nutrients and heavy metals in response to sewage sludge application to calcareous soils. *J. Agron.* **3**, 229–236 (2004).

[15] Elgarahy, A. M. et al. Biosolids management and utilizations: A review. *J. Clean. Prod.* **451**, 141974 (2024).

[16] Nazir, M. J. et al. Harnessing soil carbon sequestration to address climate change challenges in agriculture. *Soil Tillage Res.* **237**, 105959 (2024).

[17] Deng, J. et al. Enhanced sludge solid-liquid separation based on Fe2+/periodate conditioning coupled with polyoxometalates: Cell destruction and protein adsorption. *J. Environ. Manag.* **373**, 123552. 10.1016/j.jenvman.2024.123552 (2025). 10.1016/j.jenvman.2024.123552 39632306

[18] Shahbazi, F., Ghasemi, S., Sodaiezadeh, H., Ayaseh, K. & Zamani-Ahmadmahmoodi, R. The effect of sewage sludge on heavy metal concentrations in wheat plant (*Triticum aestivum* L.). *Environ. Sci. Pollut. Res.* **24**, 15634–15644 (2017). 10.1007/s11356-017-9178-z 28523618

[19] Latare, A. M., Kumar, O., Singh, S. K. & Gupta, A. Direct and residual effect of sewage sludge on yield, heavy metals content and soil fertility under rice–wheat system. *Ecol. Eng.* **69**, 17–24 (2014).

[20] Velli, P., Manolikaki, I. & Diamadopoulos, E. Effect of biochar produced from sewage sludge on tomato (*Solanum lycopersicum* L.) growth, soil chemical properties and heavy metal concentrations. *J. Environ. Manage* **297**, 113325 (2021). 34325369 10.1016/j.jenvman.2021.113325

[21] Ragonezi, C. et al. Sewage sludge fertilization—A case study of sweet potato yield and heavy metal accumulation. *Agronomy* **12**, 1902 (2022).

[22] Kumar, V. & Chopra, A. K. Agronomical performance of high yielding cultivar of eggplant (*Solanum melongena* L.) grown in sewage sludge amended soil. *Res. Agric.* **1**, 1 (2016).

[23] Tepecik, M. et al. Effects of sewage sludge on marigold (*Tagetes erecta* L.) and Garden Verbena (*Verbena hybrida*) Plants and Soil. *Kahramanmaraş Sütçü İmam Üniversitesi Tarım ve Doğa Dergisi* **26**, 161–171 (2023).

[24] Kumar, V. et al. Combined use of sewage sludge and plant growth-promoting rhizobia improves germination, biochemical response and yield of ridge gourd (*Luffa acutangula* (L.) Roxb) under field conditions. *Agriculture* **12**, 173 (2022).

[25] Gao, S. et al. Hazards of toxic metal(loid)s: Exploring the ecological and health risk in soil–crops systems with long-term sewage sludge application. *Sci. Total Environ.* **948**, 174988 (2024). 39047827 10.1016/j.scitotenv.2024.174988

[26] Sodaeizadeh, H., Karimian, A. A., Jafari, S. H. & Arani, A. M. A preliminary study on heavy metal monitoring in soil and guar (*Cyamopsis tetragonoloba*) biomass amended with sewage sludge. *Environ. Monit. Assess* **196**, 201 (2024). 38270701 10.1007/s10661-024-12337-3

[27] Al Zoubi, M. M. et al. The effect of sewage sludge on productivity of a crop rotation of wheat, maize and vetch) and heavy metals accumulation in soil and plant in aleppo governorate. *Am. Eurasian J. Agric. Environ. Sci.* **3**, 618–625 (2008).

[28] Vaca, R., Lugo, J., Martínez, R., Esteller, M. V. & Zavaleta, H. Effects of sewage sludge and sewage sludge compost amendment on soil properties and *Zea mays* L. plants (heavy metals, quality and productivity). *Revista Internacional de Contaminacion Ambiental* **27**, 303–311 (2011).

[29] Eid, E. M. et al. Effects of different sewage sludge applications on heavy metal accumulation, growth and yield of spinach (*Spinacia oleracea* L.). *Int. J. Phytoremediat.* **19**, 340–347 (2017). 10.1080/15226514.2016.1225286 27593943

[30] Chen, C. et al. The post-effects of landscape practices on spontaneous plants in urban parks. *Urban For. Urban Green.* **107**, 128744. 10.1016/j.ufug.2025.128744 (2025).

[31] Shekhawat, G. & Jana, S. Anethum graveolens: An Indian traditional medicinal herb and spice. *Pharmacogn Rev* **4**, 179 (2010). 22228959 10.4103/0973-7847.70915 PMC3249919

[32] Kaur, G. J. & Arora, D. S. Bioactive potential of *Anethum graveolens*, *Foeniculum vulgare* and *Trachyspermum ammi* belonging to the family umbelliferae—current status. *J. Med. Plants Res.* **4**, 087–094 (2010).

[33] Chahal, K., Monika Kumar, A., Bhardwaj, U. & Kaur, R. Chemistry and biological activities of *Anethum graveolens* L. (dill) essential oil: A review. *J. Pharmacogn. Phytochem.* **6**, 295–306 (2017).

[34] Lal, G., Malhotra, S. K., Lal, S. & Meena, S. S. Minor Seed Spices: Ajwain, Dill, Celery, and Aniseed. In *Handbook of Spices in India: 75 Years of Research and Development* 3505–3537 (Springer, 2023).

[35] Vincent, W. M. *The Complete Guide to Growing Healing and Medicinal Herbs : Everything You Need to Know Explained Simply*. (Atlantic Publishing Company, 2011).

[36] Gupta, R., Anwer, M. M. & Sharma, Y. K. Dill. In *Handbook of Herbs and Spices* (Woodhead Publishing, 2012).

[37] Brian, K. *Spices And Herbs: An Essential Guide To Spice Up Your Health And Flavor Your Diet*. (EWJ Publishing, 2018).

[38] Williams, V. R. *Food Cultures of Great Britain: Cuisine, Customs, and Issues*. *Food Cultures of Great Britain: Cuisine, Customs, and Issues* (2024).

[39] Meena, S., Lal, G., Mehta, R., Horticulture, K. K.-I. & 2010, U. Seed spices for home remedies. *Indian Horticulture* **55**, 1–10 (2010).

[40] Mirmolaee, S. T., Hekmatzadeh, S. F., Kazemnazhad, A., Aidenlou, F. & Shamsi, M. Evaluating the effects of Dill (*Anethum graveolens*) seed on the duration of active phase and intensity of labour pain. *J. Herb Med.* **5**, 26–29 (2015).

[41] Soil Survey Staff. *Keys to Soil Taxonomy, Twelfth Edition. Washington, DC: U.S. Department of Agriculture, Natural Resources Conservation Service, U.S. Government Printing Office*. (2014).

[42] Radford, P. J. Growth analysis formulae—Their use and abuse 1. *Crop. Sci.* **7**, 171–175 (1967).

[43] Wilke, B.-M. Determination of chemical and physical soil properties. In *Manual for Soil Analysis—Monitoring and Assessing Soil Bioremediation* (eds Margesin, R. & Schinner, F.) 47–95 (Springer, 2005).

[44] Allen, S. *Chemical Analysis of Ecological Materials* (Blackwell Scientific Publications, 1989).

[45] Eid, E. M. & Shaltout, K. H. Bioaccumulation and translocation of heavy metals by nine native plant species grown at a sewage sludge dump site. *Int. J. Phytoremediat* **18**, 1075–1085 (2016). 10.1080/15226514.2016.1183578 27184987

[46] IBM-SPSS. IBM SPSS Statistics for Windows, Version 23.0. IBM Corp., Armonk, NY. 1–3 Preprint at (2015).

[47] Aishwath, O. P. Management and utilization of degraded lands and poor quality water for production of spices, medicinal and aromatic plants. In *Horticulture Based Integrated Farming Systems* 337–350 (CRC Press, London, 2021). 10.1201/9781003245810-29.

[48] The Council of the European Communities. Council directive 86/278/EEC of 12 June 1986 on the protection of the environment, and in particular of the soil, when sewage sludge is used in agriculture. *Off. J. Euro. Commun.* **29**, 6–12 (1986).

[49] Kabata-Pendias, A. *Trace Elements in Soils and Plants* (CRC Press, 2011).

[50] Aishwath, O. P., Kumar, P., Balai, R. C. & Jalwania, R. Spices, Medicinal and Aromatic Plants in Degraded Arid and Semi-Arid Regions. In *Dryland Horticulture* 217–229 (2021).

[51] Fageria, N. K. & Moreira, A. The role of mineral nutrition on root growth of crop plants. *Adv. Agron.* **110**, 251–331 (2011).

[52] Nunes, N., Ragonezi, C., Gouveia, C. S. S. & Pinheiro de Carvalho, M. Â. A. Review of sewage sludge as a soil amendment in relation to current international guidelines: A heavy metal perspective. *Sustainability* **13**, 2317 (2021).

[53] Hafeez, A. et al. Effect of heavy metals on growth, physiological and biochemical responses of plants. In *Plants and Their Interaction to Environmental Pollution* 139–159 (Elsevier, 2023). 10.1016/B978-0-323-99978-6.00006-6.

[54] Elsayed, S. I. M., Glala, A. A., Abdalla, A. M., El-Sayed, A. E. G. A. & Darwish, M. A. Effect of biofertilizer and organic fertilization on growth, nutrient contents and fresh yield of dill (*Anethum graveolens*). *Bull. Natl. Res. Cent.* **44**, 122 (2020).

[55] Zende Bad, S. S., Rezvani Moghaddam, P., Ghorbani, R. & Khorasani, R. Effects of organic, chemical fertilizers and mycorrhizae inoculation on yield and yield components of dill (*Anethum graveolens* L.) in different cuttings. *J. Agroecol.* **10**, 621–634 (2018).

[56] Pirdashti, H., Mottaghian, A., Bahmanyar, M. A., Mottaghian, B. & Yaghubian, Y. Response of dill (*Anethum graveolens* L.) growth characters and micronutrients uptake to co-inoculation of municipal waste compost and three Trichoderma species. In *Energy, biomass and biological residues. International Conference of Agricultural Engineering—CIGR-AgEng 2012: Agriculture and Engineering for a Healthier Life, Valencia, Spain, 8–12 July 2012* (2012). 10.5555/20133223100.

[57] Fayiga, A. & Nwoke, O. Metal (Loid)s in farmland soils and strategies to reduce bioavailability. *Open J. Environ. Biol.* **2**, 009–024 (2017).

[58] Kumar, S. S., Kadier, A., Malyan, S. K., Ahmad, A. & Bishnoi, N. R. Phytoremediation and rhizoremediation: Uptake, mobilization and sequestration of heavy metals by plants. In *Plant-Microbe Interactions in Agro-Ecological Perspectives* Vol. 2 (eds Singh, D. P. et al.) 367–394 (Springer, 2017).

[59] Zheljazkov, V. D., Craker, L. E., Xing, B., Nielsen, N. E. & Wilcox, A. Aromatic plant production on metal contaminated soils. *Sci. Total Environ.* **395**, 51–62 (2008). 18353428 10.1016/j.scitotenv.2008.01.041

[60] Zheljazkov, V. D. & Warman, P. R. Application of high-Cu compost to dill and peppermint. *J. Agric. Food Chem.* **52**, 2615–2622 (2004). 15113167 10.1021/jf035137y

[61] Caunii, A. et al. Mobility of Heavy metals from soil in the two species of the aromatic plants. *Rev. Chim.* **66**, 382–386 (2015).

[62] Jalali, M. & Meyari, A. Heavy metal contents, soil-to-plant transfer factors, and associated health risks in vegetables grown in western Iran. *J. Food Compos. Anal.* **106**, 104316 (2022).

[63] Alhogbi, B. G., Al-Ansari, S. A. & El-Shahawi, M. S. A comparative study on the bioavailability and soil-to-plant transfer factors of potentially toxic element contamination in agricultural soils and their impacts: A case study of dense farmland in the western region of Saudi Arabia. *Processes* **11**, 2515 (2023).

