# Supplementary material for: Dill (Anethum graveolens L.) response to sewage sludge amendment and its impact on growth and heavy metal accumulation
Source: Sci Rep. 2025 Jul 1;15:20660. doi: 10.1038/s41598-025-97598-9 (PMC12219718; doi:10.1038/s41598-025-97598-9)
Supplement: Supplementary file 1 — Supplementary Material 1 [file 41598_2025_97598_MOESM1_ESM.docx]

**Table S1** Selected chemical properties of cultivated field soil and sewage sludge used in the pot experiment of dill plants (means ± standard error, *n* = 3)

| **Property** | **Cultivated field soil** | | **Sewage sludge** | |
| --- | --- | --- | --- | --- |
|  | **Measured value** | **Normal limit^#^** | **Measured value** | **Permissible limit**** |
| pH | 8.68 ± 0.02 | NA | 7.57 ± 0.01* | NA |
| EC (mS/cm) | 0.07 ± 0.00 | NA | 3.00 ± 0.10* | NA |
| OM (%) | 0.91 ± 0.10 | NA | 68.80 ± 0.35* | NA |
| N (mg/kg) | 779.22 ± 44.97 | NA | 3283.33 ± 28.70* | NA |
| P (mg/kg) | 595.67 ± 34.39 | NA | 259.80 ± 7.32* | NA |
| K (mg/kg) | 1482.64 ± 85.61 | NA | 1380.00 ± 16.36^ns^ | NA |
| Cd (mg/kg) | 5.91 ± 0.05 | 3 | 6.02 ± 0.74^ns^ | 20-40 |
| Co (mg/kg) | 3.16 ± 0.11 | 35 | 4.33 ± 0.10* | 10-100 |
| Cr (mg/kg) | 134.3 ± 0.7 | 125 | 2.5 ± 0.8* | 50-1750 |
| Cu (mg/kg) | 15.01 ± 0.57 | 105 | 9.98 ± 0.87* | 1000-1750 |
| Fe (mg/kg) | 2712.02 ± 50.06 | 39.2 | 1154.01 ± 24.43* | - |
| Mn (mg/kg) | 217.16 ± 10.05 | 1500-3000 | 178.33 ± 3.13* | - |
| Ni (mg/kg) | 19.41 ± 1.93 | 40 | 13.57 ± 2.72^ns^ | 300-400 |
| Pb (mg/kg) | 56.51 ± 3.04 | 160 | 127.24 ± 4.85^ns^ | 750-1200 |
| Zn (mg/kg) | 242.56 ± 5.63 | 200 | 979.00 ± 10.73* | 2500-4000 |

EC: electrical conductivity; OM: organic matter content; #: Kabata-Pendias (2011); **: Council of the European Communities (1986); the significance mark (*) indicates statistically significant differences between cultivated field soil and sewage sludge at the *p* < 0.05 level based on *t*-test; *ns*: not significant (i.e., *p* > 0.05)
